# Intelligent physical activity versus modified behavioral activation in adolescent and young adult cancer patients with psychological distress: A randomized, controlled pilot trial

**DOI:** 10.1002/cam4.5030

**Published:** 2022-07-18

**Authors:** Lijun Li, Lu Wang, Yinglong Duan, Panpan Xiao, Yi Zhou, Xiaofei Luo, Xiangyu Liu, Jianfei Xie, Andy S. K. Cheng

**Affiliations:** ^1^ The Third Xiangya Hospital of Central South University Changsha China; ^2^ Xiangya Nursing School of Central South University Changsha China; ^3^ Hunan Cancer Hospital Changsha China; ^4^ Department of Rehabilitation Sciences The Hong Kong Polytechnic University Hong Kong China

**Keywords:** adolescent and young adult, behavioral activation, cancer patients, physical activity, psychological distress, salivary cortisol

## Abstract

**Background:**

More than 80% of adolescent and young adult cancer patients (AYACPs) experienced psychological distress. Physical activity and behavioral activation are effective to relieve the psychological distress in AYACPs.

**Methods:**

Participants aged 15–39 years diagnosed with malignant tumors were included. A total of 143 eligible AYACPs were randomly assigned to three groups. The treatment‐as‐usual group (*N* = 48) received routine care, the physical activity group (*N* = 47) received 8 weeks of physical activity program based on intelligent wearable devices, and the behavioral activation group (*N* = 48) received an internet‐based modified behavioral activation program for 8 weeks. Objective outcome measures included salivary cortisol and testosterone.

**Results:**

Linear mixed‐model analyses showed significant differences between treatment‐as‐usual, physical activity and behavioral activation in salivary cortisol, as well as total scores on depression and anxiety, physical activity, self‐efficacy, and social support. Significantly stronger effect sizes for physical activity group compared with behavioral activation group were found on physical activity (*d* = 0.53) (1 week after intervention), moderate activity (*d* = 0.61), and walking activity (*d* = 0.57) (3‐month follow‐up).

**Conclusions:**

Intelligent, wearable, device‐based physical activity program is more effective in alleviating anxiety and depression, reducing saliva cortisol, and improving physical activity in AYACPs than internet‐based modified behavioral activation program. Intelligent, wearable, device‐based physical activity program can reduce the time cost of AYACPs to ensure that the intervention is carried out.

## Graphical abstract

This study combined the objective indicators (salivary cortisol) to explore the effect of an intelligent wearable device‐based physical activity program and internet‐based modified behavioral activation program on alleviating psychological distress in adolescent and young adult cancer patients (AYACPs) by a three‐arm RCT. The results demonstrated that an intelligent wearable device‐based physical activity program and internet‐based modified behavioral activation program can effectively alleviate anxiety and depression in AYACPs. Including objective evaluation metrics in psychological distress research is feasible.

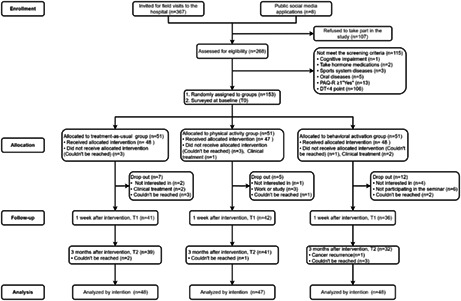

## 1 INTRODUCTION

The National Comprehensive Cancer Network (NCCN) has classified cancer patients who were diagnosed between the ages of 15 and 39 years old as adolescent and young adult cancer patients (AYACPs).^1^ With advances in medical technology and cancer screening, AYACPs have an 85% 5‐year survival rate.^2^ The prevalence and severity of psychological distress are higher and more common among AYACPs.^3^, ^4^ According to the Clinical Practice Guidelines for the Management of Psychological Distress in Oncology developed by NCCN, psychological distress in cancer patients was defined as a multifactorial emotional experience that includes psychological (e.g., cognitive, behavioral, emotional), social, and/or spiritual substance. These experiences may affect patients' ability to cope effectively with the disease, its signs and symptoms, and treatment.^5^ Adolescence and young adulthood are important transitional stages of life with many fundamental developmental changes. Being diagnosed with cancer during this time may pose unique developmental challenges for them at this particularly vulnerable point, like developing social growth and role changes.^6^ AYACPs suffer from diverse psychological distress, such as anxiety, depression, and cognitive impairment.^7^ They differ in biological, epidemiological and clinical outcomes from other age groups of cancer patients.^8^

Physical activity programs, recommended by American Cancer Society, can effectively promote the recovery of somatic functions, relieve anxiety, depression, and cancer‐caused fatigue and improve the quality of life of AYACPs.^9^ The Canadian Mood and Anxiety Treatment Network recommend physical activity program for the treatment of mild to moderate depression.^10^ It can prevent a high incidence of negative behavior in AYACP‐sedentary behavior, which increases the risk of chronic disease and secondary tumors.^11^ On the other hand, young people are the mainstream consumers of electronic products and social media, and interventions with smart wearable devices can be used to monitor and supervise patients' exercise.^12^

Behavioral activation program were originally designed to treat depression by reducing avoidance and withdrawal behaviors, increasing involvement in daily activities and enabling positive reinforcement in the patient's life, and returning the patient to normal life.^13^ Behavioral activation program outperforms other psychotherapeutic approaches in the treatment of mild to moderate depression.^14^ The behavioral activation component of cognitive therapy alone can also have comparable efficacy with the overall cognitive therapy.^15^ It is more durable, economical, and has fewer side effects than drug therapy.^16^ This program based on cognitive program with light physical activity is effective in alleviating depression.^17^ Compared with traditional behavioral activation, internet‐based modified behavioral activation programs may address the geographical and psychological isolation experienced by some AYACPs. Behavioral activation may also support AYACPs' use of coping skills that may have a long‐term impact on their quality of life.^18^

The diagnosis of cancer is a stressful event that may lead to disturbances in the balance of the neuroendocrine and immune systems.^19^ Stress activates the hypothalamic–pituitary–adrenocortical (HPA) axis, causing a large secretion of stress hormones (cortisol and catecholamines). The HPA axis is composed of a hormonal cascade: psychological and physiological stresses lead to the secretion of corticotropin‐releasing hormone (CRH) by the hypothalamus. CRH leads to the secretion of adrenocorticotropic hormone (ACTH) by pituitary corticotropin cells. In turn, ACTH causes the adrenal cortex to secrete cortisol. Cortisol exerts negative feedback on the secretion of these two upstream hormones. The HPA axis is dysregulated in a wide range of physiological and pathological conditions (e.g., cessation of alcohol consumption and childbirth).^20^ The HPA axis interacts with the synthesis and secretion of salivary cortisol, and its concentration can be a useful indicator to observe the response of the HPA axis.^21^ Increased cortisol concentration is a physiological manifestation of distress.^22^ Psychological distress may alter the activity of the HPA axis in cancer patients, leading to abnormal cortisol secretion and increasing the risk of immune dysfunction and cancer progression.^23^ Salivary cortisol also is a more accurate measure of psychological distress in cancer patients than total plasma cortisol.^24^ In addition, free testosterone is associated with changes in psychological distress,^25^ changes in salivary testosterone also reflect changes in total blood testosterone.^26^

Social support is a personal psychosocial resource and correlates with psychological distress in AYACPs.^4^ A previous study showed that psychological distress and self‐efficacy are factors that affect the quality of life during cancer treatment.^27^ Sleep quality is a risk factor for poor psychological functioning in AYACPs and is strongly associated with mental health functioning.^28^

**FIGURE 1:**
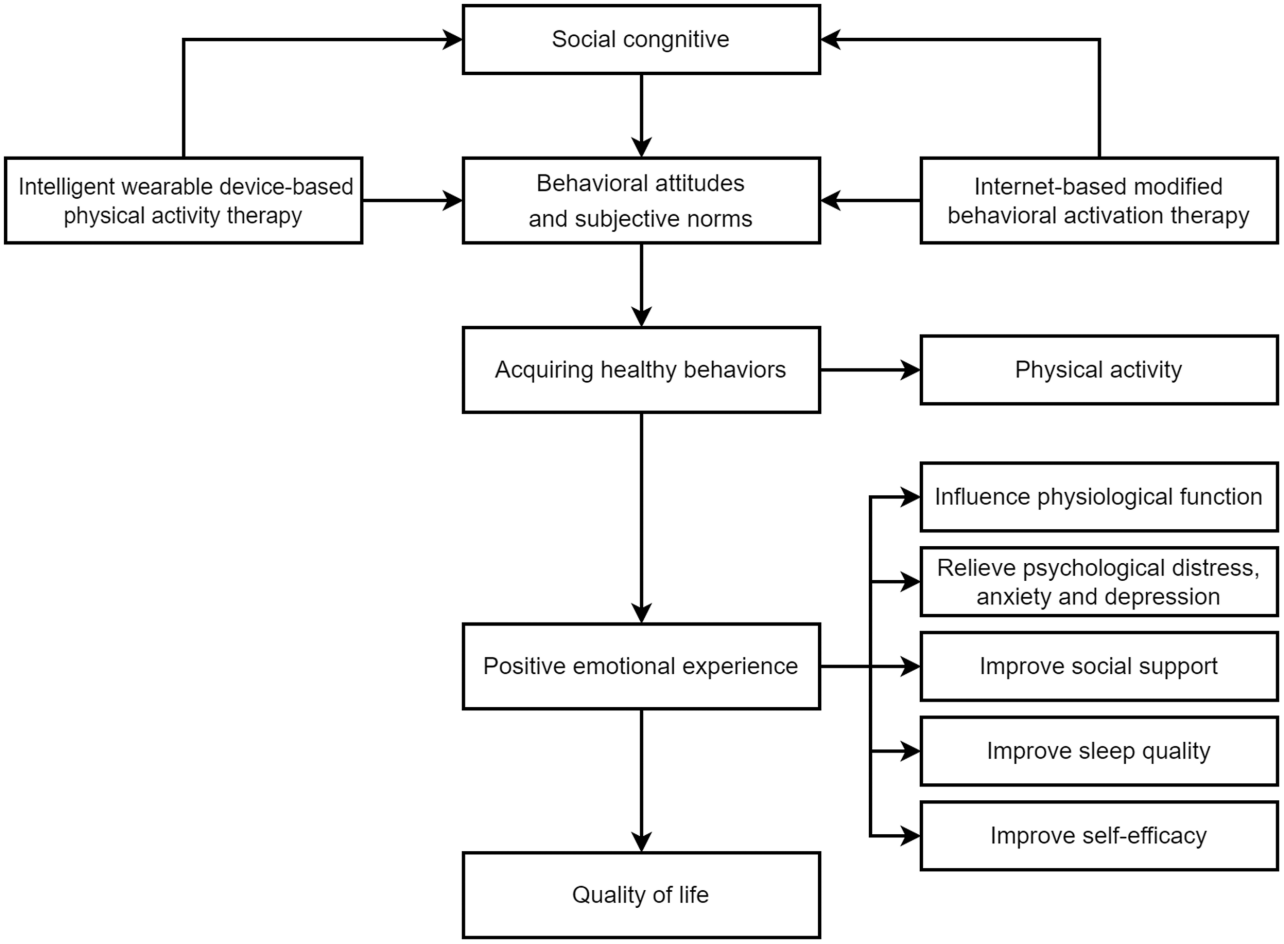
The theoretical framework.

The theoretical framework of this study is social cognition theory and planned behavior theory.^29^, ^30^ As shown in Figure 1, physical activity and behavioral activation program directly or indirectly influence individuals' behavioral attitudes and subjective norms through their social cognition, thus enabling them to acquire healthy behaviors, increase their physical activity, and shape healthy behaviors to help them acquire positive emotional experiences, improve their physiological functions, alleviate their psychological distress, anxiety and depression levels, and improve social support, sleep quality, and self‐efficacy, thus improving their quality of life.

Therefore, this study aimed to (1) explore the association between salivary cortisol and psychological distress, (2) evaluate the effects and compare the difference between the physical activity and behavioral activation program on AYACPs' psychological distress, and (3) understand the effects of these interventions at varying time points.

## 2 METHODS

### 2.1 Design and procedures

This was a randomized controlled, three‐arm trial with repeated measures conducted between March 2018 and August 2019. In this randomized controlled trial, the physical activity group was compared with the behavioral activation group and with the treatment‐as‐usual group to expect a significant reduction in salivary cortisol levels, anxiety, and depression at both follow‐up time points. Three occasions of assessment were conducted: at baseline (T_0_), 1 week after the intervention (T_1_), and 3 months after intervention (T_2_). The treatment‐as‐usual group received routine nursing psychological care. The two experimental groups were (1) the physical activity group, which participated in physical activity based on intelligent wearable devices plus treatment‐as‐usual and (2) the behavioral activation group, which received an online behavioral activation program plus treatment‐as‐usual.

**FIGURE 2:**
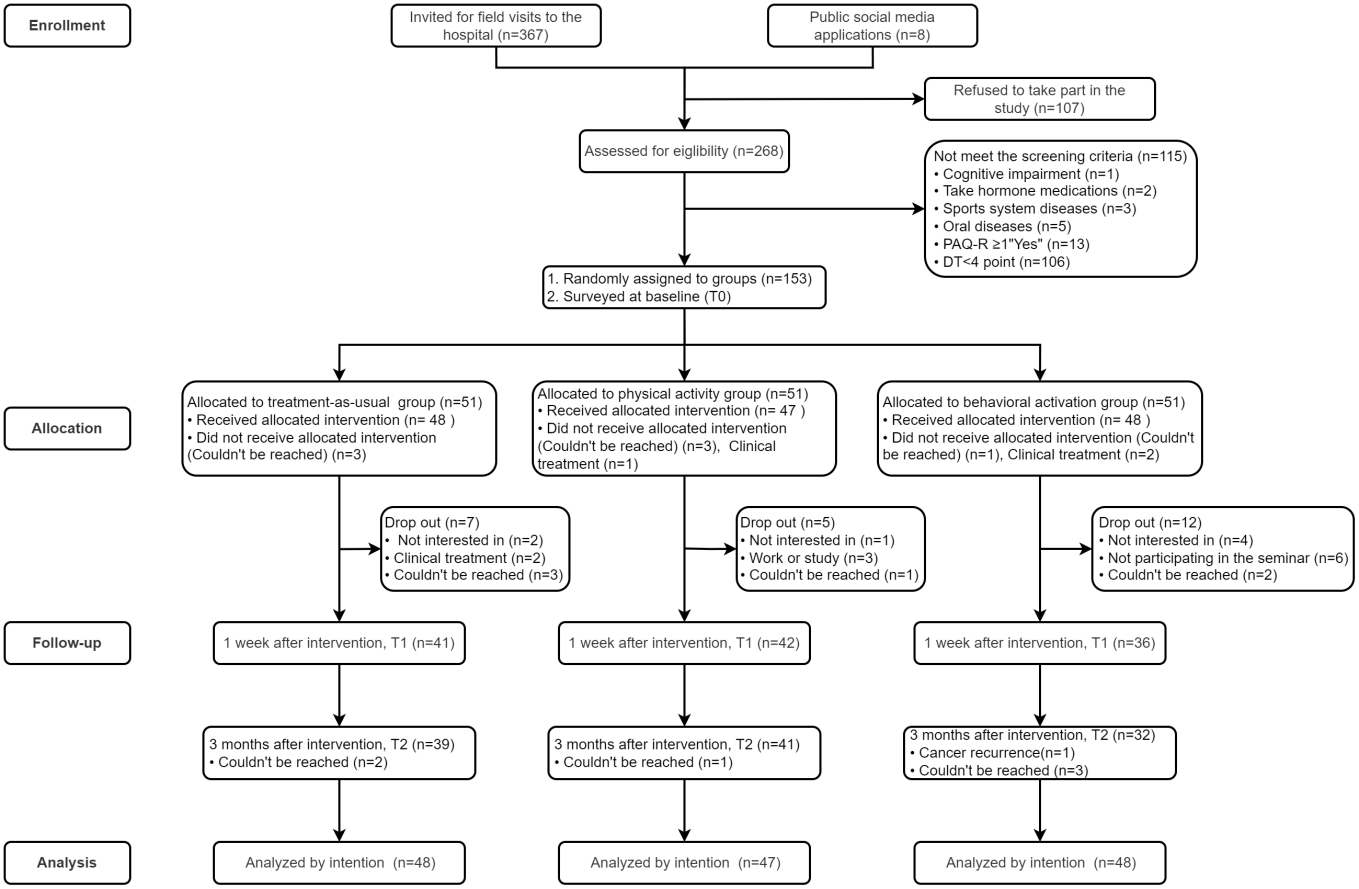
CONSORT flowchart depicting recruitment. DT, distress thermometer; PAR‐Q, Physical Activity Readiness Questionnaire.

AYA cancer survivors were recruited during field visits to the hospitals and through social media. Details of the study were posted on social media (anticancer community website, post‐bar, WeChat, etc.). The Distress Thermometer (DT)^31^ and Physical Activity Readiness Questionnaire (PAR‐Q)^32^ were used to screen the eligibility of the participants. The DT and PAR‐Q were used to measure the psychological distress and safety among AYACPs participating in exercise interventions, respectively. After the screening, participants were randomly assigned to the physical activity group, behavioral activation group, or treatment‐as‐usual group by a random number table. Each participant was numbered according to the order of admission (Figure 2).

### 2.2 Participants

The inclusion criteria were as follows: (1) 15–39 years of age; (2) diagnosed with cancer in the past 5 years; (3) adults voluntarily participate in the study and sign an informed consent form; minors must be willing and have their guardians' consent and co‐sign an informed consent form; and (4) Chinese speaker. Participants were excluded if they (1) had an existing mental illness or other systemic diseases; (2) had a communication disorder; (3) exhibited drug or alcohol dependency; (4) suffering from other oral type diseases such as oral mucosal disease, periodontal disease, and dental caries; and (4) had a DT score <4 points (no psychological distress) or if the participant answered “yes” to any of the PAR‐Q questions.

### 2.3 Interventions

Before beginning the study, each potential participant was seen individually either in person or by video call, particularly for those who were invited through social media.

#### 2.3.1 Treatment‐as‐usual group

Participants in the treatment‐as‐usual group received usual treatment and follow‐up, which included providing disease‐related knowledge, training in self‐care skills, and routine nursing psychological care by clinical nurses.

#### 2.3.2 Physical activity group

In addition to usual treatment and follow‐up, each participant in the physical activity group was given an actigraph and an exercise instruction manual. The manual explained how to use the exercise bracelet, the method, intensity and frequency of exercise, how to prevent exercise injuries, precautions during exercise, and the start and end time nodes of the whole intervention. The actigraph was used to ensure that participants engaged for the expected. According to the American Cancer Society's recommendation of “moderate‐intensity exercise for at least 150 minutes a week”,^33^ we designed the physical activity plan for AYACPs. WeChat group managers posted 1–2 cancer‐ or exercise‐related questions on social media for participants to answer and discuss each week to promote intragroup interaction (Table 1).

**TABLE 1:** Comparison of intervention contents among different intervention groups

	Treatment‐as‐usual group	Physical activity group	Behavioral activation group
Expectation	Reduced psychological distress of participants	Moderate intensity exercise of at least 150 min per week to relieve participants' psychological distress	Relieving participants' psychological distress by implementing online behavioral activation therapy
Intervention forms	Handbook	Handbook, smart sports bracelet, and WeChat group	Handbook and WeChat group
Intervention contents			
Behavioral ability	—	Walking exercise according to 8‐week exercise prescription	Participate in eight seminars and complete personalized daily activities
Self‐efficacy	—	The smart sports bracelet supporting application provides feedback, according to the guidance to set individual exercise goals, check the comparison chart of exercise data for effect feedback, and obtain positive motivation through health consultation	Weekly seminars to increase a sense of control and positive coping through positive reinforcement; compare the differences in the daily activity registers to discover changes in your daily activities and changes in your life; break down difficult tasks and cascade them to completion
Self‐monitor	To enable patients to participate in their own treatment and nursing as much as possible and to achieve maximum self‐care	Smart sports bracelet tracks and records sports data, participant sports diary	Complete weekly assignments
Social support	—	Participants' family and friends join the WeChat group, which pushes links, articles, and campaign reminders to encourage communication and experience sharing within the group	Complete weekly assignments under the supervision of friends and family, push links, articles and exercise reminders by WeChat, encourage group communication and experience sharing
Intervention period	Eight weeks	Eight weeks	Eight weeks
Length and frequency of each intervention	Floating	For the first 2 weeks, walking three times a week (20–30 min/time) In weeks 3–4, walking 4 times a week (30–40 min/time) For the last 4 weeks, walk five times per five times per week (30–40 min/time)	Eight times, 120–180 min per session

#### 2.3.3 Behavioral activation group

Other than usual treatment and follow‐up, participants in the behavioral activation group were further divided into smaller groups of 6–10 participants each, and a 120‐ to 180‐min online video seminar was offered to them once a week. Based on the modified behavioral activation treatment suggested by Xie et al.^34^ an 8‐week progressive content was provided for the participants. WeChat group managers posted 1–2 cancer‐ or psychological distress‐related questions on social media for participants to answer and discuss each week to promote interaction within the group (see Supporting information).

In addition, for the intervention groups, participants' relatives or friends were supported to join the WeChat group to understand the intervention dynamics and provide social support and assistance to the AYACPs.

### 2.4 Measures

#### 2.4.1 Demographic data

Demographic information, including gender, age, educational background, marital status, monthly income, time for diagnosis, living situation, occupation, residence, complications, treatment program, cancer classifications, and cancer staging, was collected.

#### 2.4.2 Primary outcomes

##### Salivary biomarkers

Since the levels of cortisol and testosterone will influence the state of the HPA axis, they can be used as effective indicators to observe the HPA axis response.^35^ Moreover, the concentration of salivary‐free cortisol is close to that of blood‐free cortisol, and the change in salivary testosterone also reflects the change in total blood testosterone.^36^ Therefore, an enzyme‐linked immunosorbent assay (ELISA) was performed to measure salivary cortisol and salivary testosterone by ELISA kits (IBL International).

Because the biosynthesis and secretion of cortisol are regulated by the HPA axis and have a fixed diurnal pattern of change, reaching a maximum in the early morning and a minimum at midnight, all biochemical samples were collected in the morning from 7:00 to 8:00 a.m. and completed within the time window of the psychological test to avoid the influence of time on the assessment index.

##### The Hospital Anxiety and Depression Scale

The Hospital Anxiety and Depression Scale (HADS) was developed to assess anxiety and depression experienced in the previous week.^37^ It contains 14 items with a 4‐point Likert scale that are divided into two dimensions: anxiety and depression. Depression and anxiety scores are summed separately. The scores for each dimension range from 0 to 21. Higher values indicate a higher level of anxiety or depression; scores above 9 indicate the presence of anxiety or depression.^38^

#### 2.4.3 Secondary outcome measures

The International Physical Activity Questionnaire (IPAQ) was used to measure the level of physical activity, which was formulated by the World Health Organization in 1998. There are 27 items in total, which are composed of seven parts: occupation, family, transportation, physical activities related to leisure and entertainment, and sedentary. Divided into three kinds of physical activities: high‐intensity, moderate, and walking.^39^

The Social Support Rating Scale (SSRS) was used to measure social support through three subscales: objective support (three items), subjective support (four items), and support utilization (three items).^40^

The General Self‐Efficacy Scale (GSES) reflects optimistic self‐belief.^41^ Zhang and Schwarzer developed the Chinese version of the GSES to evaluate general self‐efficacy in the general population.^42^

The Pittsburgh Sleep Quality Index (PSQI) was used to assess subjective sleep quality in the recent month.^43^ It consists of 19 self‐rated items and seven dimensions: subjective sleep quality, sleep latency, sleep duration, habitual sleep efficiency, sleep disturbances, hypnotic use, and daytime dysfunction.^44^

All these measures were selected based on their satisfactory construct validity and reliability and their availability in Chinese. Three occasions of assessment were conducted: at baseline (T_0_), 1 week after the intervention (T_1_), and 3 months after intervention (T_2_).

### 2.5 Sample size and allocation

Based on repeated measures analysis of variance and a similar previous study that assessed changes in HADS scores,^45^ effect sizes of 0.25, *α* = 0.05, and 1‐*β* = 0.8 were set. As a result, the calculated total sample size was 108. We anticipated that as many as 20% of the participants may be lost during follow‐up; therefore, the minimum sample size in this study was 135. Participants were numbered according to the sequence of enrollment, and participants with equipped numbers were informed to third‐party personnel, who entered the computer according to the sequence. Participants were randomly grouped by the random sequence generated by SPSS 24.0. The third‐party personnel were unaware of the purpose and process of this study and were not concerned with the findings of this study, ensuring that the randomized grouping was allocated hide. All participants did not know which group they were allocated to before the intervention, although the researchers knew.

### 2.6 Ethical statement

This study was conducted according to the guidelines of the Declaration of Helsinki, and ethical approval was granted by the Ethics Committee of The Third Xiangya Hospital of Central South University (no. 2015‐S032). Registration number: ChiCTR2100049229. All participants signed written consent prior to randomization into different groups.

### 2.7 Statistical analysis

All statistical analyses were performed by SPSS 24.0 software for Windows (IBM Corp). Descriptive statistics, ANOVAs, Kruskal–Wallis tests, Tamhane tests, and χ^2^ tests were used to determine whether participants' characteristics (sociodemographic and clinical) were different across experimental conditions. A predefined alpha of 0.05 was used. The corrected statistical significance was *α* (0.05)/*k*. Missing data were filled in by intention‐to‐treat analysis. Linear mixed models (LMMs), with fixed effects and interaction effects for group and time, assessment and their two‐way interaction, as well as a random intercept for randomization group and participants (nested within the randomization group), were used to investigate differences in the course of the outcome measures between the three groups. Between‐group and within‐group differences were assessed by LMM and independent sample *t*‐tests with post hoc analysis, respectively. For the latter, Bonferroni's correction was employed for multiple testing. Effect sizes (Cohen's *d*) were calculated by dividing the difference in change between groups by the pooled standard deviation at separate assessment time points (1 week post‐intervention and 3 months post‐intervention). Effect sizes of 0.2 were categorized as small, 0.5 as medium, and 0.8 as large.^46^

## 3 RESULTS

### 3.1 Recruitment and participants' characteristics at baseline

A total of 375 potential participants were enrolled for initial screening, and 153 of them were eligible and randomly assigned to three groups with each of 51 samples. Because of clinical treatment and the inability to contact the few eligible participants, the final number of participants in the treatment‐as‐usual group, physical activity group and behavioral activation group were 48, 47 and 48, respectively. One week after the intervention, 143 questionnaires were distributed, but only 119 valid questionnaires were returned. Finally, 3 months after the intervention, 119 questionnaires were issued, and 112 valid questionnaires were received. Therefore, the attrition rate of this study was 26.8%. Figure 2 presents the reasons for loss to follow‐up in each group. At baseline, participants' demographic and clinical characteristics of the three groups were not significantly different, including cancer, psychological, physical activity variables, and biochemical indicators (*p* > 0.05) (Table 2).

**TABLE 2:** Demographic and medical characteristics in the three groups (*N* = 143)

Demographic and medical conditions	Treatment‐as‐usual (*N* = 48)	Physical activity (*N* = 47)	Behavioral activation (*N* = 48)	*F*/*χ* ^2^/*H*	*p*
Age, year	31.21 ± 5.539	28.40 ± 6.275	29.21 ± 7.246	2.431	0.092
Gender					
Male	12 (25.0)	9 (19.1)	13 (27.1)	2.836	0.585
Female	36 (75.0)	38 (80.9)	35 (72.9)		
Education background					
Elementary school and below	13 (27.1)	10 (21.3)	8 (16.7)	2.441	0.295
Middle school	19 (39.6)	15 (31.9)	18 (37.5)		
High school	8 (16.7)	8 (17.0)	11 (22.8)		
Junior college or university	7 (14.6)	14 (29.8)	11 (22.9)		
Master and above	1 (2.1)	0	0		
Marital status					
Single	5 (10.4)	8 (17.0)	9 (18.8)	1.012	0.603
Married	42 (87.5)	36 (76.6)	38 (79.2)		
Divorced/widowed	1 (2.1)	3 (6.4)	1 (2.1)		
Monthly income, RMB					
<500	11 (22.9)	14 (29.8)	6 (12.5)	1.740	0.419
500–1000	15 (31.3)	6 (12.8)	13 (27.1)		
1000–3000	16 (33.3)	13 (27.7)	15 (31.3)		
3000–5000	5 (10.4)	9 (19.1)	8 (16.7)		
>5000	1 (2.1)	5 (10.6)	6 (12.5)		
Live with family					
No	2 (4.2)	4 (8.5)	0 (0.0)	4.249	0.119
Yes	46 (95.8)	43 (91.5)	48 (100.0)		
Residence					
Rural	29 (60.4)	29 (61.7)	27 (56.3)	1.012	0.603
Town	10 (20.8)	7 (14.9)	7 (14.6)		
County	4 (8.3)	2 (4.3)	4 (8.3)		
Urban	5 (10.4)	9 (19.1)	10 (20.8)		
Time from diagnosis					
Within 3 months	14 (29.2)	16 (34.0)	17 (35.4)	1.768	0.413
3–6 months	15 (31.3)	15 (31.9)	18 (37.5)		
6–12 months	8 (16.7)	5 (10.6)	7 (14.6)		
1–3 years	6 (12.5)	8 (17.0)	6 (12.5)		
More than 3 years	14 (29.2)	3 (6.4)	0		
Complication					
No	34 (70.8)	40 (85.1)	36 (75.0)	2.876	0.237
Yes	14 (29.2)	7 (14.9)	12 (25.0)		
Treatments					
Only surgery	9 (18.8)	6 (12.8)	5 (10.4)	4.937	0.294
Surgery + radiotherapy	28 (58.3)	28 (59.6)	23 (47.9)		
Surgery + radiotherapy + chemotherapy	11 (22.9)	13 (27.7)	20 (41.7)		
Cancer classifications					
Lung cancer	17 (35.4)	10 (21.3)	16 (33.3)	4.678	0.586
Gynecological tumors^a^	10 (20.8)	10 (21.3)	12 (25.0)		
Breast cancer	14 (29.2)	14 (29.8)	12 (25.0)		
Others^b^	7 (14.6)	13 (27.7)	8 (16.7)		
Cancer staging					
I‐IIa	11 (22.9)	18 (38.3)	18 (37.5)	6.850	0.106
IIb‐IIIa	27 (56.3)	16 (34.0)	16 (33.3)		
IIIb	10 (20.8)	13 (27.7)	14 (29.2)		

^a^ Gynecological Tumors including cervical, ovarian and uterine tumor.

^b^ Other types of cancer including nasopharyngeal cancer and colorectal cancer.

### 3.2 Impact of physical activity and behavioral activation intervention for AYACPs

Table 3 shows the results of LMM for variables between groups and at different assessment time points. Statistically significant differences were found between and within groups on the salivary cortisol, the level of physical activity (IPAQ) (i.e., total score, moderate and walking activity), and social support (SSRS) (*p* < 0.001). Psychological distress (HADS) (i.e., total score and anxiety) and self‐efficacy were statistically significantly different between the three groups and at different assessment time points (*p* < 0.001). The depression (subscale of HADS) and sleep quality (PSQI) indicated statistically significant differences across different assessment time points (*p* < 0.001). However, there was no statistically significant difference in testosterone between and within the three groups across different assessment time points.

**TABLE 3:** Linear mixed models analyzing intervention outcome (*N* = 143)

Variables	Time	Treatment‐as‐usual (*n* = 48)	Physical activity (*n* = 47)	Behavioral activation (*n* = 48)	Fixed effect	*F*	*p*
		Mean ± SD					
Salivary cortisol	T_0_	6.55 ± 0.30	6.47 ± 0.29	6.49 ± 0.30	Group	63.977	<0.001*
	T_1_	5.37 ± 0.38	4.46 ± 0.27	4.35 ± 0.50	Time	722.933	<0.001*
	T_2_	6.19 ± 0.49	5.84 ± 0.54	5.84 ± 0.45	Group × Time	20.812	<0.001*
Testosterone	T_0_	86.76 ± 123.82	86.29 ± 94.91	87.22 ± 110.17	Group	0.052	0.949
	T_1_	92.18 ± 119.60	99.41 ± 129.56	97.56 ± 117.50	Time	0.424	0.655
	T_2_	94.94 ± 84.53	100.34 ± 112.83	98.28 ± 95.62	Group × Time	0.016	0.999
HADS							
Total	T_0_	12.29 ± 3.98	11.23 ± 3.34	11.63 ± 3.26	Group	8.397	<0.001*
	T_1_	10.98 ± 2.87	9.55 ± 2.36	9.84 ± 2.57	Time	14.398	<0.001*
	T_2_	11.03 ± 3.08	9.23 ± 2.82	9.54 ± 3.19	Group × Time	0.261	0.903
Anxiety	T_0_	4.88 ± 1.75	4.49 ± 1.69	4.54 ± 1.88	Group	12.137	<0.001*
	T_1_	4.36 ± 2.00	3.35 ± 1.23	3.54 ± 1.24	Time	20.067	<0.001*
	T_2_	4.24 ± 1.25	3.21 ± 0.78	3.37 ± 1.12	Group × Time	0.817	0.515
Depression	T_0_	7.42 ± 3.48	6.74 ± 3.14	7.00 ± 3.24	Group	1.966	0.141
	T_1_	6.62 ± 1.72	6.60 ± 1.83	6.30 ± 2.21	Time	3.165	0.043*
	T_2_	6.79 ± 2.54	6.02 ± 2.87	6.17 ± 3.01	Group × Time	0.066	0.993
IPAQ							
Total	T_0_	303.81 ± 200.37	305.77 ± 182.63	305.56 ± 190.24	Group	57.956	<0.001*
	T_1_	367.76 ± 198.69	818.00 ± 362.98	644.14 ± 266.47	Time	79.188	<0.001*
	T_2_	387.48 ± 221.81	884.18 ± 317.06	646.98 ± 238.94	Group × Time	14.511	<0.001*
Intensity of activity	T_0_	56.30 ± 33.97	57.03 ± 34.29	56.92 ± 33.92	Group	2.788	0.063
	T_1_	57.24 ± 28.72	71.69 ± 36.56	68.08 ± 34.12	Time	2.745	0.065
	T_2_	55.76 ± 28.61	67.10 ± 33.30	62.10 ± 25.26	Group × Time	0.643	0.632
Moderate activity	T_0_	71.63 ± 67.29	72.40 ± 67.45	72.12 ± 66.83	Group	27.930	<0.001*
	T_1_	100.34 ± 74.71	273.49 ± 218.925	218.08 ± 163.82	Time	46.212	<0.001*
	T_2_	111.57 ± 80.258	309.32 ± 223.59	225.23 ± 149.44	Group × Time	7.081	<0.001*
Walking activity	T_0_	175.89 ± 180.13	176.35 ± 183.22	176.51 ± 179.91	Group	27.376	<0.001*
	T_1_	202.18 ± 176.02	472.82 ± 289.47	359.98 ± 228.96	Time	33.435	<0.001*
	T_2_	220.15 ± 175.29	507.76 ± 263.05	359.65 ± 206.34	Group × Time	6.890	<0.001*
Self‐efficacy							
Total	T_0_	2.36 ± 0.70	2.30 ± 0.66	2.25 ± 0.77	Group	6.594	<0.001*
	T_1_	3.50 ± 0.53	3.40 ± 0.65	3.15 ± 0.42	Time	120.786	<0.001*
	T_2_	3.14 ± 0.45	2.87 ± 0.44	2.86 ± 0.39	Group × Time	1.082	0.365
Social support							
Total	T_0_	36.27 ± 3.99	35.87 ± 5.48	36.50 ± 4.08	Group	33.967	<0.001*
	T_1_	43.93 ± 6.21	51.37 ± 4.83	51.25 ± 5.24	Time	211.906	<0.001*
	T_2_	41.71 ± 5.53	48.34 ± 7.37	48.05 ± 5.54	Group × Time	8.996	<0.001*
PSQI							
Total	T_0_	6.42 ± 2.850	6.32 ± 3.108	6.46 ± 2.858	Group	3.014	0.050
	T_1_	5.91 ± 2.213	4.96 ± 2.259	5.33 ± 1.878	Time	9.347	<0.001*
	T_2_	5.88 ± 2.303	4.86 ± 2.339	5.05 ± 1.591	Group × Time	0.658	0.621

Abbreviations: HADS, Hospital Anxiety and Depression Scale; IPAQ, International Physical Activity Questionnaire; PSQI, Pittsburgh Sleep Quality Index.

* *p* < 0.05.

Post hoc analyses revealed that for participants in the physical activity group, there were significant intervention effects in all variables except for depression and testosterone compared with the treatment‐as‐usual group (*p* < 0.05). Compared with treatment‐as‐usual groups, there were significantly better intervention effects for decreasing saliva cortical penetration concentration, relieving psychological distress, and anxiety, improving physical activity, moderate and walking activity, social support, and general self‐efficacy in the physical activity group (*p* < 0.05). We also found that physical activity, moderate and walking activities performed finer in the physical activity group than in the behavioral activation group (*p* < 0.05) (Table 4).

**TABLE 4:** Linear mixed‐model analyses: differences between two study arms (*N* = 143)

	Physical activity—treatment as usual	Physical activity—behavioral activation	Behavioral activation—treatment as usual
	*p*	*p*	*p*
Salivary cortisol	<0.001*	0.396	<0.001*
Testosterone	0.758	0.940	0.815
HADS			
Total	<0.001*	0.361	0.003*
Anxiety	<0.001*	0.442	<0.001*
Depression	0.056	0.599	0.163
IPAQ			
Total	<0.001*	<0.001*	<0.001*
Intensity of activity	0.021*	0.447	0.119
Moderate activity	<0.001*	0.004*	<0.001*
Walking activity	<0.001*	0.001*	<0.001*
Self‐efficacy			
Total	0.031*	0.155	<0.001*
Social support			
Total	<0.001*	0.911	<0.001*
PSQI			
Total	0.016*	0.420	0.108

Abbreviations: HADS, Hospital Anxiety and Depression Scale; IPAQ, International Physical Activity Questionnaire; PSQI, Pittsburgh Sleep Quality Index.

* *p* < 0.05.

### 3.3 The effect size between the three groups at different time points

Table 5 shows the intervention effects at baseline (T_0_) and 1 week (T_1_) and 3 months (T_2_) after the intervention. When comparing the physical activity group with the treatment‐as‐usual group between baseline and 1 week after the intervention, large effect sizes were found for the primary outcome measure of salivary cortisol (*d* = 1.93, *p* < 0.001), secondary outcome measure of total physical activity (*d* = 1.47, *p* < 0.001), moderate activity (*d* = 0.96, *p* < 0.001), walking activity (*d* = 1.21, *p* < 0.001), and social support (*d* = 1.34, *p* < 0.001). However, high‐intensity activity was observed at a moderate effect size (*d* = 0.64, *p* = 0.009). Three months after the intervention, a moderate effect size was still found for salivary cortisol (*d* = 0.54, *p* = 0.006), but total physical activity (*d* = 1.71, *p* < 0.001), moderate activity (*d* = 1.15, *p* < 0.001), walking activity (*d* = 1.08, *p* < 0.001), and social support (*d* = 0.97, *p* < 0.001) were able to sustain a large effect size between baseline and 3 months after the intervention.

**TABLE 5:** Intervention effects at 1‐week (T_1_), 3‐month (T_2_) follow‐up compared with baseline (T_0_) (*N* = 143)

Variables	T_1_ vs T_0_			T_2_ vs T_0_			T_1_ vs T_2_		
	PA‐TAU	PA‐BA	BA‐TAU	PA‐TAU	PA‐BA	BA‐TAU	PA‐TAU	PA‐BA	BA‐TAU
	^*a*^d, *p*								
Salivary cortisol	1.93, <0.001	0.26, ns	1.71, <0.001	0.54, 0.006	0.12, ns	0.55, 0.014	1.20, <0.001	0.12, ns	0.96, <0.001
IPAQ									
Total	1.47, <0.001	0.53, 0.011	0.88, <0.001	1.71, <0.001	0.79, 0.001	0.92, <0.001	0.12, ns	0.18, ns	0.06, ns
High intensity	0.64, 0.009	0.22, ns	0.45, 0.039	0.48, 0.041	0.24, ns	0.31, ns	0.10, ns	0.05, ns	0.17, ns
Moderate	0.96, <0.001	0.27, ns	0.74, 0.001	1.15, <0.001	0.61, 0.044	0.87, <0.001	0.14, ns	0.11, ns	0.04, ns
Walking	1.21, <0.001	0.44, 0.033	0.56, 0.007	1.08, <0.001	0.57, 0.005	0.45, 0.016	0.05, ns	0.14, ns	0.07, ns
Social support									
Total	1.34, <0.001	0.13, ns	1.26, <0.001	0.97, <0.001	0.25, ns	1.06, <0.001	0.09, ns	0.06, ns	0.12, ns

Abbreviations: BA, behavioral activation; IPAQ, International Physical Activity Questionnaire; PA, physical activity; TAU, treatment‐as‐usual.

^a^ *d* corresponds to between group difference score of Cohen's *d*.

Likewise, when comparing the behavioral activation group with the treatment‐as‐usual group between baseline and 1 week after the intervention, large effect sizes were found for salivary cortisol (*d* = 1.71, *p* < 0.001), total physical activity (*d* = 0.88, *p* < 0.001), and social support (*d* = 1.26, *p* < 0.001). Three months after the intervention, a moderate effect size was still found for salivary cortisol (*d* = 0.55, *p* = 0.006). Similar to the physical activity group, total physical activity (*d* = 0.92, *p* < 0.001), moderate activity (*d* = 0.87, *p* < 0.001), and social support (*d* = 1.06, *p* < 0.001) were able to sustain a large effect size between baseline and 3 months after the intervention.

For the comparison of the physical activity group and behavioral activation group, moderate effect sizes were observed for total physical activity at T_1_ (*d* = 0.53, *p* = 0.011) and T_2_ (*d* = 0.79, *p* = 0.001). Finally, moderate effect sizes were observed for moderate activity (*d* = 0.61, *p* = 0.044) and walking activity (*d* = 0.57, *p* = 0.005) at T_2_.

## 4 DISCUSSION

In this study, the physical activity group was designed to alleviate participants' psychological distress by increasing their exercise levels, while the behavioral activation group was based on a cognitive program with light activity. This study used a rigorous design to compare the effects of physical activity and behavioral activation programs on psychological distress in AYACPs and clearly described the intervention steps and follow‐up. Modified Internet‐based behavioral activation reduces the geographic isolation of participants.^18^ Physical activity intervention was combined with a smart wearable device that provides real‐time data and monitoring.^47^ The attrition rates suggested that both interventions are acceptable for AYACPs and easier to implement.^48^

The results of this study showed that there was a statistically significant difference between the three groups in AYACP salivary cortisol levels (*p* < 0.001). It was significantly lower in the physical activity and behavioral activation groups 1 week after the interventions. This result was similar to that of Rao et al.,^49^ who showed that morning cortisol levels were significantly lower in cancer patients who underwent exercise intervention. Physical activity will induce fluctuations in serum glucocorticoids,^50^ and its activation of the HPA axis has been verified in animal experiments.^51^ Physical activity modulates HPA axis function by increasing adrenal sensitivity and promoting increased levels of the pro‐ACTH‐releasing factor, thereby reducing psychological distress‐related behaviors and emotions.^50^ This study showed not only the physiological effects of an exercise‐based intervention but also the feasibility of including objective evaluation metrics (e.g., salivary cortisol) in psychological distress research.

In this study, 8 weeks of an intelligent wearable device‐based physical activity program presented a higher total IPAQ score for AYACPs than a web‐based modified behavioral activation program (*p* < 0.001), and there was also a moderate effect size of the difference between the two intervention groups (*d* > 0.5) at T_2_. This result suggested that compared with behavioral activation program, physical activity programs were more likely to promote high levels of physical activity in AYACPs. Although there was no significant difference between the two intervention groups in terms of relieving anxiety and depression, reducing morning salivary cortisol secretion, or improving quality of life, social support, and sleep quality, the findings suggest a tendency for the intervention effect of physical activity program to be better to that of behavioral activation program. This is consistent with the study by Soucy et al.,^17^ which suggested that physical activity program may be an important component and logical basis for depression interventions based on activating behavioral changes. The dominance exhibited by physical activity program, albeit weak, has tremendous application in alleviating psychological distress in AYACPs.

The HADS, IPAQ, SSRS, GSES, and PSQI scores of the physical activity group at 3 months after the intervention were significantly higher than the preintervention scores, with statistically significant differences (*p* < 0.05), verifying that the 8‐week intelligent wearable device‐based physical activity program was not only effective in relieving patients' anxiety and depression and improving their physical activity, social support, and self‐efficacy but also in maintaining the intervention effect for at least 3 months. Participation in regular physical activities can foster psychological growth experiences after cancer diagnosis and treatment.^52^ There is a significant relationship between social support and physical activity participation.^53^ A mediating effect of physical activity self‐efficacy on the relationship between symptom distress and exercise participation in AYACPs who are undergoing cancer treatment.^54^ Previous studies mostly showed the maintenance effect of physical activity program 2 months after the intervention.^55^ Despite the small difference in the extension time, it still provides a realistic basis for the long‐term maintenance of the exercise program intervention effect.

The physical activity group had the lowest attrition rate (12.8%) compared with the other two groups. Unlike the physical activity group, which used intelligent wearable devices, the behavioral activation group in this study adopted a web‐based modified behavioral activation program that required intensive participation in sessions and had more therapeutic components and was thus time‐consuming. Compared with children and the elderly, the AYACP group is more geographically mobile,^56^ and they survive with cancer for a longer period while being affected by work, school, and treatment. They may not be able to focus their time to participate in psychotherapy^57^ to alleviate their psychological distress. Physical activity program, can reduce the time cost of AYACPs to ensure that the intervention is carried out. As a result, it can have a long‐term interventional effect.

### 4.1 Strengths and limitations

The strengths of this study were the specific focus on AYACPs, an important life development stage of cancer patients. Furthermore, we explored the advantage of implementing physical activity program with intelligent wearable devices in AYACPs and used objective outcomes (biochemical indicators) to assess the treatment effect. Nevertheless, there were some limitations in this study. First, we only set up the treatment‐as‐usual, physical activity, and behavioral activation groups, separately. But there is no physical activity combined behavioral activation group, confounding bias may exist in this study. Second, this study was conducted in Hunan Province, central China, and thus, its epidemiological representativeness may be challenged. Finally, the present study reassessed outcomes until 3 months after the intervention. Therefore, future studies are recommended to extend the follow‐up period to determine the maintenance effects of the interventions.

## 5 CONCLUSIONS

To conclude, the results of this study suggested that both intelligent wearable‐based physical activity programs and web‐based modified behavioral activation programs were effective in relieving anxiety and depression, reducing morning salivary cortisol, and improving physical activity, self‐efficacy, social support, and sleep quality in AYACPs. Intelligent wearable device‐based physical activity program has advantages over web‐based modified behavioral activation programs in improving physical activity in youth.

## AUTHOR CONTRIBUTIONS

All authors certify that they have participated in this work. The manuscript was conceptualized and supervised by Lijun Li and Lu Wang, with data curation, original draft writing, and review and editing by Lijun Li. Jianfei Xie contributed to methodology, formal analysis, project administration, funding acquisition. Lu Wang assisted with data curation. Lijun Li, Lu Wang, Yinglong Duan, Panpan Xiao, Yi Zhou, Xiaofei Luo and Xiangyu Liu all contributed to investigation. Jianfei Xie and Andy SK Cheng participated in review and editing. All authors read and approved the final manuscript.

## FUNDING INFORMATION

This study funded by The Wisdom Accumulation and Talent Cultivation Project of the Third Xiangya Hospital of Central South University (no. YX202006) and The National Natural Science Foundation of China (no. 82073409).

## CONFLICT OF INTEREST

The authors declare no conflicts of interest.

## ETHICAL STATEMENT

This study was conducted according to the guidelines of the Declaration of Helsinki, and ethical approval was granted by the Ethics Committee of The Third Xiangya Hospital of Central South University (no. 2015‐S032). Registration number: ChiCTR2100049229.

## Supporting information


Figure S1

## Data Availability

The data that support the findings of this study are available from the corresponding author upon reasonable request.
