# Supplementary figures and images for: Intelligent physical activity versus modified behavioral activation in adolescent and young adult cancer patients with psychological distress: A randomized, controlled pilot trial
Source: Cancer Med. 2022 Jul 18;12(2):1935–48. doi: 10.1002/cam4.5030 (PMC9883549; doi:10.1002/cam4.5030)

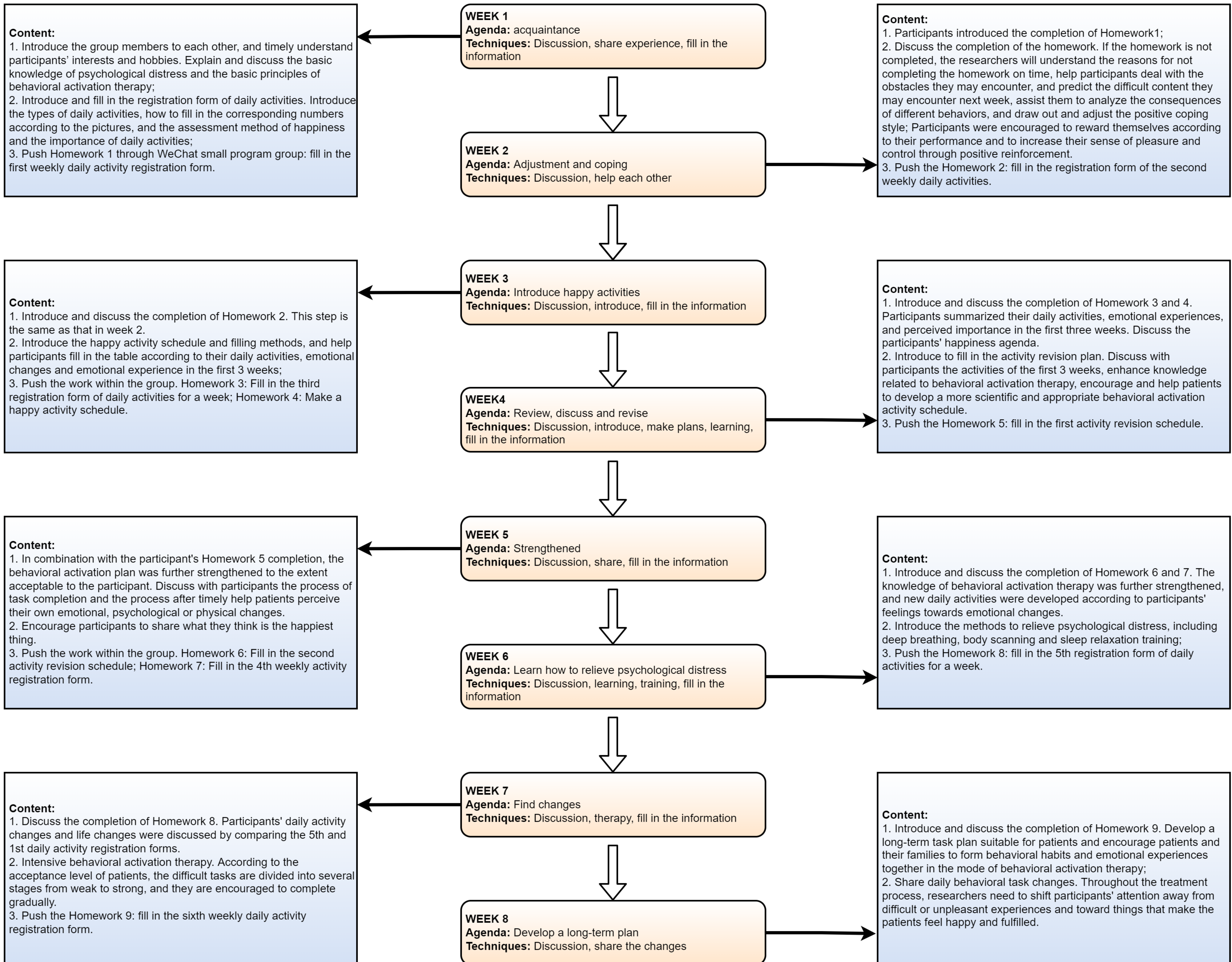

Supplement: Supplementary file 1 — Figure S1 [file CAM4-12-1935-s001.pdf]
